# Creativity, Proactive Personality, and Entrepreneurial Intention: The Role of Entrepreneurial Alertness

**DOI:** 10.3389/fpsyg.2018.00951

**Published:** 2018-06-14

**Authors:** Rui Hu, Li Wang, Wei Zhang, Peng Bin

**Affiliations:** ^1^College of Public Administration, Huazhong Agricultural University, Wuhan, China; ^2^Institute of International and Comparative Education, Beijing Normal University, Beijing, China; ^3^School of Education Science, Jiangsu Normal University, Xuzhou, China

**Keywords:** creativity, proactive personality, entrepreneurial alertness, entrepreneurial intention, mediation effect

## Abstract

This study examines the extent to which entrepreneurial alertness mediates the effects of students’ proactive personalities and creativity on entrepreneurial intention. Drawing on a field survey of 735 Chinese undergraduates at 26 universities, this study provides evidence for the argument that entrepreneurial alertness has a fully mediation effect on the relationship between creativity, a proactive personality, and entrepreneurial intention. The findings shed light on the mechanisms that underpin entrepreneurial alertness and contribute to the literature on key elements of the entrepreneurial process.

## Introduction

Given rising unemployment rates and the richer opportunities offered by rapid globalization and marketization, more and more college students are opting to start their own businesses. A large body of empirical studies has tried to ascertain the extent to which entrepreneurship contributes to economic growth by creating new jobs and encouraging innovation. Given that researchers have highlighted the importance of intentions as antecedents of behavior in recent years (Shook et al., 2003), investigating the factors that influence individual intention to pursue entrepreneurship could accelerate the development of the entrepreneurial process. Such research could shape individual entrepreneurial intention, providing crucial support for both theory and practice. Although the relationship between different factors and entrepreneurial intention has been widely studied, the decision processes that facilitate individual entrepreneurial behavior remain an open issue (Markmaan et al., 2002; Zampetakis, 2008).

Scholars have debated the vital role played by entrepreneurial alertness in the entrepreneurial process. “The value of examining entrepreneurial alertness is that it concerns the individual’s awareness, assessment, and orientation toward uncertainties and changes in the external environment and context—beyond the within-person, internal issue of identity” (Uy et al., 2015). The influences of proactive personality and creativity on entrepreneurial desirable and intention have also been widely studied (Crant, 1996; Zampetakis, 2008). Hansen et al. (2011) have argued that creativity and proactive personality are the main factors influencing entrepreneurial intention because they are closely linked with identifying opportunities; they serve an important function in encouraging new enterprises. However, to the best of our knowledge, the relationships between entrepreneurial alertness, creativity, proactive personality, and entrepreneurial intentions are still under-explored; few studies have tested the indirect effect of creativity or proactive personality on entrepreneurial intention outside Western culture. It is therefore important to connect entrepreneurial alertness and proactive personality with entrepreneurial intention to provide new theoretical and practical insights. To tackle this timely issue, we have used a university student sample from the Chinese mainland to examine whether, and the extent to which, creativity and proactive personality are associated with entrepreneurial alertness and intention.

The paper is structured as follows. Section “Literature Review and Hypothesis Development” briefly reviews previous studies that touch on the relationship between proactive personality, creativity, and entrepreneurial intention. Section “Aims and Hypotheses” introduces our hypotheses. The methodology and data are presented in section “Materials and Methods.” Section “Results” reveals the relationships among the targeted variables. The paper concludes with a discussion of the contributions and limitations of this study (see section “Discussion and Conclusion”).

## Literature Review and Hypothesis Development

### Entrepreneurial Alertness

One of the most significant topics in the field of entrepreneurship is the awareness of entrepreneurial opportunities, previously addressed by Campos (2016). Israel Kirzner’s theory of entrepreneurial alertness has added value to the research on opportunity identification. Kirzner (1973) was the first to define the role played by entrepreneurial alertness in helping entrepreneurs become more aware of new opportunities and use limited clues in different ways. In further research, Kirzner (1979) has defined entrepreneurial alertness as an individual’s ability to perceive new opportunities that have hitherto been overlooked by others; more specifically, alertness can be described as: “a motivated propensity of man to formulate an image of the future” (Kirzner, 1985). In accordance with Kirzner’s research, Baron and Ensley (2006) have argued that alert individuals “identify new solutions to market and customer needs in existing information, and to imagine new products and services that do not currently exist” (Baron and Ensley, 2006). A central study by McMullen and Shepherd (2006) has confirmed that, “To act on the possibility that one has identified an opportunity worth pursuing” is the heart of being an entrepreneur (McMullen and Shepherd, 2006). Gaglio and Katz (2001) have asserted that a high level of entrepreneurial alertness leads to acute sensitivity to one’s surroundings, a mental framework that helps entrepreneurs adjust to the current situation. Based on previous research, Tang et al. (2012) have produced a 13-item model, which theoretically and empirically divides entrepreneurial alertness into three distinct factors: (a) “scanning and search,” (b) “association and connection,” and (c) “evaluation and judgment.” Tang’s instrument is well-validated and has strong reliability, providing researchers with a valuable tool for probing the entire entrepreneurial opportunity-development process, including antecedents and outcomes.

In the Chinese context, Li et al. (2015) and Hu and Ye (2017) have studied Chinese university students, concluding that entrepreneurial alertness directly and significantly predicts opportunity recognition. The goal of alertness research is to discover not only the antecedents of alertness, but also its outcomes (Kirzner, 2009). The present study aims to reveal whether, and the extent to which, entrepreneurial alertness mediates the effects of creativity and a proactive personality on entrepreneurial intention.

### Creativity, Proactive Personality, and Entrepreneurial Alertness

Creativity significantly influences an individual’s entrepreneurial alertness. Creativity, which refers to the development of novel and useful ideas, is closely related to innovation and generally studied at the individual level (Amabile, 1996; Zampetakis, 2008). Furthermore, the interaction between an individual and his or her environment will yield creativity (Hunter et al., 2007). Factors such as an individual’s intrinsic motivation, personality, knowledge, cognitive style, and social background can play important roles in the cultivation of creativity (Woodman and Schoenfeldt, 1990; Sternberg and Lubart, 1995; Amabile, 1996).

Creativity has become a central theme in entrepreneurial process research. The relationship between creativity and enterprise has been confirmed by researchers: novel ideas are produced when access to information is clear and free (Biraglia and Kadile, 2017); such new ideas exemplify the nature of creativity and lead to new entrepreneurial ventures (McMullan and Kenworthy, 2015). Creativity is a key element at the start of the entrepreneurial process, since it contributes to the design of new products and services (Heinonen et al., 2011; Gielnik et al., 2012). Feldman and Bolino (2000) have argued that individuals with higher perceived creativity are more likely to build up their own businesses; this confirms the findings of Sternberg and Lubart (1999), who have noted that entrepreneurship is, to some extent, the result of creativity.

Given the importance of identifying entrepreneurial opportunities in the field of entrepreneurship, experts maintain that alertness about opportunities is important, not only for innovation behavior (Obschonka et al., 2017), but also for successful entrepreneurial behavior (Baron, 2006). Kirzner (2009) believes that entrepreneurial alertness involves creative behavior. This, to some extent, confirms the conclusion drawn by Ardichvili et al. (2003) that a conceptual link exists between creativity and entrepreneurial alertness. Empirical studies have confirmed that creative activities involving the use of new communication and Internet technologies may also predict entrepreneurial alertness. Campos (2016) and Obschonka et al. (2017) have empirically confirmed that creativity has a significant relationship with entrepreneurial alertness, particularly in the dimensions of scanning and searching. Campos (2016) has used a moderated mediation model to identify a positive correlation between creativity and entrepreneurial alertness. Alertness requires a creative act, which may influence the further development and improvement of entrepreneurial opportunities (Baron, 2004; Kirzner, 2009). Thus, creativity is associated with entrepreneurial alertness.

The proactive personality is also associated with entrepreneurial alertness. “Proactivity refers to active attempts made by the individual to effect changes in his or her environment” (Zampetakis, 2008). According to Bateman and Crant (1993), people with proactive personalities tend to take the initiative to influence and even to significantly change the environment. In other words, having a proactive personality can help an individual release situational pressures, identify opportunities for advantage, make proactive moves, and thereby influence the environment to create meaningful changes (Bateman and Crant, 1993). Alongside previous research on environmental adaptability (Crant, 1995, 2000) and proactivity, additional empirical studies have confirmed that proactive individuals are more likely to achieve success at work and more positively attuned to the need for dominance, achievement, self-confidence, and conscientiousness (Claes et al., 2005). Previous studies have identified a positive relationship between proactive personality and entrepreneurship (e.g., Becherer and Maurer, 1999; Kickul and Zaper, 2000). For instance, Becherer and Maurer (1999) have related proactivity to starting rather than buying or inheriting a business, as well as to the number of businesses started.

Proactive personality is seen as the crucial antecedent of entrepreneurial alertness to opportunities because opportunity identification is an important aspect of individual initiative (Ardichvili et al., 2003; Tang et al., 2012). Using a survey that researched a diverse group of undergraduate students from Singapore, Uy et al. (2015) have shown that entrepreneurial alertness partially mediates the relationship between a proactive personality and a boundless career mindset, and confirming that personality traits, such as proactive personality, are connected to entrepreneurial alertness. Obschonka et al. (2017), using two-wave longitudinal data from high schools in Helsinki, Finland, has confirmed that entrepreneurial alertness is predicted by different underlying competencies; furthermore, both creativity and proactivity improve the link between personality and entrepreneurial alertness.

### Entrepreneurial Alertness and Entrepreneurial Intention

Entrepreneurial alertness is closely associated with individual entrepreneurial intention. Entrepreneurial intention is “a self-acknowledged conviction by a person that they intend to set up a new business venture and consciously plan to do so at some point in the future” (Thompson, 2009). It constitutes a more or less concrete plan to prepare for, and then ultimately start, an entrepreneurial career of one’s own in the future (Obschonka et al., 2017). Entrepreneurial intention plays a crucial role in shaping an individual’s entrepreneurial behaviors (Markmaan et al., 2002), “the stronger a person’s intention to engage in a specific behavior, the more likely it is that the actual behavior will be performed” (Ajzen, 1991).

The connection between entrepreneurial alertness and entrepreneurial intention has been empirically confirmed by McMullen and Shepherd (2006), who have argued that entrepreneurial alertness improves the judgment and opportunity identification of individuals, helping to form entrepreneurial intention and future business behavior. In a Chinese context, Li et al. (2015) have confirmed that entrepreneurial alertness is an essential strength for entrepreneurs because it directly predicts opportunity recognition. Guided by social cognitive theory, Hu and Ye (2017), using a sample of 364 Chinese students majoring in sports, have confirmed that both entrepreneurial alertness and entrepreneurial self-efficacy are key cognitive predictors of entrepreneurial intention.

Having reviewed the existing literature, we therefore raise our hypotheses in section “Aims and Hypotheses.”

## Aims and Hypotheses

As discussed in the Sections “Introduction” and “Literature Review and Hypothesis Development,” the relationships between entrepreneurial alertness, creativity, proactive personality, and entrepreneurial intentions remain under-explored. Therefore, this study had the objective to fill the gap in the literature in two ways. First, resorting to a nationally representative survey of entrepreneurship of Chinese college students, this study aims to investigate the relationships among creativity, proactive personality, entrepreneurial alertness and entrepreneurial intention in the Asian culture. Second, the current study mainly focuses on how the entrepreneurial alertness mediates the effects of students’ proactive personalities and creativity on entrepreneurial intention. This mediation effect of entrepreneurial alertness had not yet been investigated in the context of this theory. Accordingly, this study raises the following hypotheses:

(1) There is a positive relationship between creativity and entrepreneurial alertness (Hypothesis 1).
(2) There is a positive relationship between proactive personality and entrepreneurial alertness (Hypothesis 2).
(3) There is a positive relationship between entrepreneurial alertness and entrepreneurial intention (Hypothesis 3).
(4) Cumulatively, the predictions above suggest the role and relevance of mediation: creativity influences students’ entrepreneurial alertness, which in turn positively affects entrepreneurial intention (Hypothesis 4a). Similarly, a proactive personality influences students’ entrepreneurial alertness, which in turn positively affects entrepreneurial intention (Hypothesis 4b).

**FIGURE 1:**
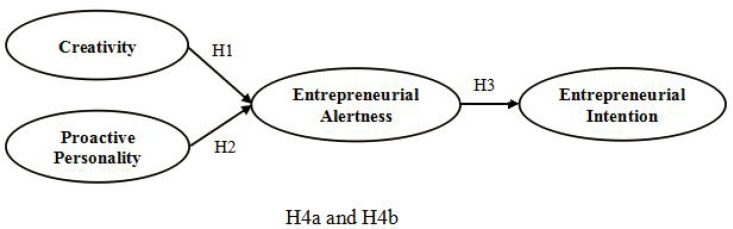
The proposed structural relationships between creativity, proactive personality, entrepreneurial alertness, and entrepreneurial intention.

To examine the four hypotheses, the current study extends from the previous wisdom and designs the model with the mediator. The operationalization of the mediating model in current study is described in **Figure 1**.

## Materials and Methods

### Participants

The sample was drawn from a nationally representative survey. A total of 857 undergraduate students from 26 universities volunteered to participate in this survey. Of the initial dataset, 122 respondents were eliminated due to incomplete or questionable response patterns (e.g., selecting “5” as a response across an entire section of the survey, which included reverse-worded items); this study ultimately produced 735 valid samples. To keep the sample unbiased, the survey selected respondents studying a wide range of fields: a fair distribution across 19 areas of specialization guaranteed a representative sample of students, enabling us to analyze the entrepreneurial intentions of college students in studying fields.

The participants were majoring in Agricultural Science (6%), Animal Science (7%), Aquaculture (7%), Biological Sciences (7%), Business Administration (5%), Chemistry (5%), Computing Science (4%), Economics (5%), Environmental Science (7%), Food Science (5%), Horticulture (7%), Law (5%), Marketing Management (4%), Mechanics (5%), Public Administration (4%), Sociology (5%), and Tea Study (5%). The participants were aged 18–22 with a mean of 20.02 years (*SD* = 3.11). Of the total sample, 59.6% (*n* = 438) were males and 40.4% (*n* = 297) were females, with mean ages of 20.77 years (*SD* = 5.75) and 19.76 years (*SD* = 1.60), respectively. The distribution by academic year was as follows: 48.2% were 1^st^ year college students (*n* = 354); 33.3% were 2^nd^ year college students (*n* = 245); 6.8% were 3^rd^ year college students (*n* = 50); 7.6% were 4^th^ year college students (*n* = 56); 3.5% were 1^st^ year graduate students (*n* = 26); and 0.5% were 2^nd^ year graduate students (*n* = 4).

### Instruments

All of the independent and dependent variables were evaluated using self-report measures based on multi-item scales. Responses across all dimensions and items were made using five-point Likert-type scales, ranging from 1 (strongly disagree) to 5 (strongly agree). All of the items were translated into Mandarin by a native speaker; they were re-translated into English by other bilingual individuals to guarantee that the scales had similar quality and connotations. The instruments and some items in the constructs are outlined below.

#### Proactive Personality

The proactive personality scale used in this study was the six-item scale adopted by Bateman and Crant (1993). The original scales were those of Claes et al. (2005). The proactive personality scale included items such as, “Regardless of the odds, if I believe in something, I will make it happen.” The Cronbach’s alpha was 0.845. All of the items accounted for 78.08% of the variance.

#### Creativity

Creativity was measured using an eight-item scale developed by Zhou and George (2001). The items referred to the production of useful and creative ideas (Zampetakis, 2008). Examples included the following: “I develop adequate plans and schedules for the implementation of new ideas” and “I suggest new ways to increase the quality of project assignments.” The Cronbach’s alpha for all eight items was 0.919. All of the items accounted for 64.02% of the variance.

#### Entrepreneurial Alertness

Entrepreneurial alertness was tested using Tang et al.’s (2012) scale, which categorizes 13 items into three factors: scanning and search, association and connection, and evaluation and judgment. Examples included the following: “I have frequent interactions with others to acquire new information” (scanning and search), “I often see connections between previously unconnected domains of information” (association and connection), and “When facing multiple opportunities, I am able to select the good ones” (evaluation and judgment). The alpha coefficient of the general alertness factor was 0.879. All of the items accounted for 64.15% of the variance with each of the three factors accounting for 45.89, 11.14, and 7.11%, respectively. The mean for entrepreneurial alertness was 3.48 (*SD* = 0.6).

#### Entrepreneurial Intention

We assessed entrepreneurial intention using scales developed by Krueger et al. (2000). Although Krueger et al.’s (2000) scale included nine factors, this study dropped three factors and used a six-item scale to suit the Chinese context. For example, “I intend to start my own business in the near future.” The alpha coefficient of the entrepreneurial intention scale was 0.915. All of the items explained 70.39% of the variance. The mean of entrepreneurial intentions was 2.74 (*SD* = 0.78).

#### Control Variables

We controlled for the students’ gender, age, and entrepreneurial role models (whether a parent or relative was an entrepreneur). According to Díaz-García and Jiménez-Moreno (2010), male students’ express higher entrepreneurial intention than female students. Demographic factors appear to significantly affect entrepreneurial intention (Hockerts, 2017).

### Procedure

To reduce the sample selection bias, our survey used various classrooms and collected the questionnaires randomly. Informed consent was obtained from all participants. Participants were asked to complete a questionnaire that included 5 sections and 27 questions, covering entrepreneurial intention, entrepreneurial alertness, creativity, and proactive personality, as well as demographic features. The participants were asked whether they agreed or disagreed with each question. This study did not involve any potential risk for the participants. They were also informed that participation was voluntary and anonymous; the data were protected by applicable legislation. Four members of the research team traveled to the various universities to conduct this survey.

#### Data Analysis

The current study analyzed the relationships between each variable, using Pearson’s correlation coefficient and SPSS version 22. We tested the theoretical model in **Figure 1** using structural equation modeling (SEM) via AMOS 17.0 and evaluated model fit using the Chi-square statistic, the Chi-square-to-degrees-of-freedom ratio, the goodness-of-fit index (GFI), the comparative fit index (CFI), the Tucker-Lewis index (TLI), and the root-mean square error of approximation (RMSEA). Researchers have suggested that levels of 0.90 or higher for GFI, CFI, and TLI (the closer to 1, the better the index) and levels of 0.08 or lower for RMSEA (the closer to 0, the better the RMSEA) indicate that a model fits the data appropriately (Lance et al., 2007; Kim et al., 2009).

We then tested the research hypotheses by examining whether each structural path was statistically significant. In addition, we tested the mediation effects of entrepreneurial alertness on the relationship between creativity and proactive personality and entrepreneurial intention using a Chi-square difference test (Anderson and Gerbing, 1988; Hui et al., 1999). Specifically, we compared the mediated model (as proposed in **Figure 1**) to a full model that included direct effects. We then tested whether the χ^2^ difference between the two models was significant or not. A non-significant χ^2^ difference indicates a full mediation effect (Hui et al., 1999; Kim et al., 2009). For the mediation test, the following three conditions had to be met: (1) predictive and mediating variables have significant effects on the outcome variable; (2) predictive variables have significant effects on the mediating variables; (3) the effect of predictive variables on the outcome variable tends to weaken when mediating variables are added, while the effect of the mediating variables on the outcome variable remain significant (Wen et al., 2016). Finally, we used bootstrapping procedures (re-sampled 1000 times, with the percentile method used to create 95% confidence intervals) to further confirm the mediation effects.

## Results

### Descriptive Statistics

A confirmatory factor analysis (CFA) was conducted to assess the discriminant validity of the measures. Our hypothesized four-factor structure was confirmed by the CFA results. The four-factor model had significantly lower Chi-square statistics [χ^2^(113) = 622.185, *p* < 0.01] than a one-factor model with all items loaded on a single construct [χ^2^(122) = 2926.02, *p* < 0.01]. This is confirmed by the fact that other indices of the four-factor model (RMSEA = 0.078; GFI = 0.902; CFI = 0.922; TLI = 0.906) fit better than those of the one-factor model (RMSEA = 0.177; GFI = 0.596; CFI = 0.57; TLI = 0.521). As expected, each item loaded on its hypothesized factor with large and significant loadings; each construct extracted a variance that was larger than the highest variance it shared with any other construct, thus providing support for discriminant validity.

**Table 1** presents the descriptive statistics, reliability estimates, and correlations for all measures. It can be clearly observed that the reliabilities of all variables are in the reasonable range (0.79–0.88). A strong correlation can be observed both between entrepreneurial intention and its antecedents and between these antecedents. The main statistics in **Table 1** deliver the following messages: (1) both creativity and proactive personality are significantly related to entrepreneurial alertness and entrepreneurial intention; (2) the control variables (gender and business-owning parent) are both significantly related to entrepreneurial intention. These findings suggest that students who are more creative, proactive, and alert are more likely to start their own businesses.

**Table 1:** Descriptive statistics and correlations for the total sample.

	*M*	*SD*	1	2	3	4	5	6	7
(1) Gender^a^	1.6	0.5	-						
(2) Age	20.02	3.11	-0.100^∗∗^	-					
(3) Parent owns a business^b^	1.61	0.49	0.019	-0.061	-				
(4) Creativity	3.49	0.65	-0.028	-0.055	-0.056	(0.87)			
(5) Proactive personality	3.49	0.6	-0.028	-0.034	-0.007	0.548^∗∗^	(0.85)		
(6) Entrepreneurial alertness	3.47	0.6	-0.065	-0.045	-0.065	0.656^∗∗^	0.639^∗∗^	(0.79)	
(7) Entrepreneurial intention	2.74	0.78	-0.151^∗∗^	0.028	-0.102^∗∗^	0.355^∗∗^	0.332^∗∗^	0.324^∗∗^	(0.88)


**N* = 735. Internal reliabilities are in parenthesis.*

*^*a*^Gender is coded-1: male 2: female.*

*^*b*^Parent owning a business is coded-1: yes 2: no.*

*^∗∗^*p* < 0.01.*

### Assessment of the Structural Model

To test the hypotheses, we carried out a structural equation analysis of the relationships between creativity, proactive personality, entrepreneurial alertness, and entrepreneurial intention. In particular, we examined whether creativity and a proactive personality were both positively related to entrepreneurial alertness (Hypotheses 1 and 2), whether entrepreneurial alertness was significantly related to entrepreneurial intention (Hypothesis 3), and whether or not the effect of entrepreneurial intention was mediated by entrepreneurial alertness (Hypotheses 4a and 4b), as shown in **Figure 1**. Model 1 represents the predicted mediating model. We drew paths from creativity and proactive personality to entrepreneurial alertness and from entrepreneurial alertness to entrepreneurial intention. As shown in **Table 2**, Model 1 represented a good fit to the data (RMESA = 0.065, GFI = 0.91, CFI = 0.922, TLI = 0.91).

**Table 2:** Comparison of structural equation models (Models 1 and 2).

Model	χ^2^	*df*	Δχ^2^ (Δ*df*)	RMESA	GFI	CFI	TLI
Model 1	676.428^∗∗∗^	165		0.065	0.91	0.922	0.91
Model 2	675.227^∗∗∗^	163	1.201 (2)	0.065	0.91	0.922	0.909


**N* = 735, χ^*2*^: Chi-square statistic.*

*Model 1: Creativity + proactive personality→ entrepreneurial alertness→ entrepreneurial intention (including gender, age, and parent owning a business as control variables).*

*Model 2: Creativity + proactive personality*^®^* entrepreneurial alertness*^®^* entrepreneurial intention (additional line: creativity + proactive personality*^®^* entrepreneurial intention; including gender, age and parent owning a business as control variables).*

*^∗∗∗^*p* < 0.001.*

We adopted a second model to further test the mediation effect. As shown in **Table 2**, Model 2 includes two additional direct paths: from creativity and proactive personality to entrepreneurial intentions. Consistent with Model 1, the control variables (gender, age, and parent owns a business) are included in Model 2. As shown in **Table 2**, the difference in Chi-square between Model 1 and Model 2 was not significant (Δχ^2^ = 1.201, Δ*df* = 2). The other indices were almost unaffected by including the two additional paths in the model (RMESA = 0.065, GFI = 0.91, CFI = 0.922, TLI = 0.91). The insignificant Δχ^2^/Δ*df* also suggests a complete mediation effect of entrepreneurial alertness. According to Kelloway (1998), the parsimonious model should be adopted if similar results are obtained from two competing models; for this reason, Model 1 has been adopted in current study.

To better illustrate the mediation effect of entrepreneurial alertness, a bootstrapping regression has been carried out to further demonstrate Model 1. **Table 3** shows the results of the bootstrapping regression, demonstrating that the value of the mediation effect of entrepreneurial alertness is 0.232, between creativity and entrepreneurial intention, in the confidence interval (0.164, 0.307) and 0.253, between proactive personality and entrepreneurial intention, in the confidence interval (0.187, 0.345). This suggests that entrepreneurial alertness plays a significant intermediary role between independent variables (creativity and proactive personality) and entrepreneurial intention.

**Table 3:** Bootstrapping regression results for creativity and proactive personality mediated by entrepreneurial alertness.

Variables	Estimate	*SE*	Lower 2.5%	Upper 2.5%	*p*
Creativity → Entrepreneurial	0.232	0.036	0.164	0.307	0.002
Alertness → Entrepreneurial intention					
Proactive personality → Entrepreneurial	0.253	0.04	0.187	0.345	0.001
Alertness → Entrepreneurial intention					


*Dependent variable: entrepreneurial intention (EI).*

**N* = 735, bootstrap sample size = 1000.*

**FIGURE 2:**
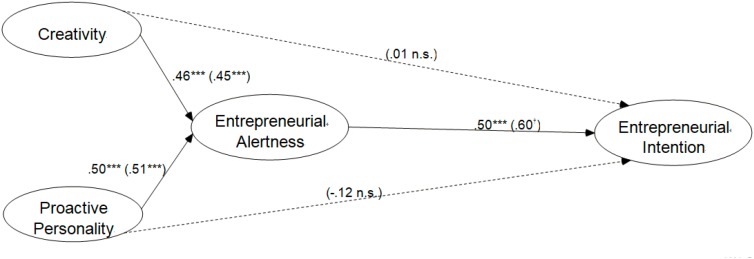
Estimated path coefficients of the structural equation model. The numbers represent the beta coefficients for Model 1. The beta coefficients for Model 2 are in parentheses. ^†^Marginal significant. ^∗∗∗^*p* < 0.001.

We predicted that creativity would be positively associated with entrepreneurial alertness. The significant parameter estimate was consistent with this, as shown in **Figure 2** (β = 0.46, *p* < 0.001). **Figure 2** also shows that proactive personality was positively and significantly related to entrepreneurial alertness (β = 0.50, *p* < 0.001). In addition, the parameter estimate displayed in **Figure 2** indicates that entrepreneurial alertness was positively and significantly related to entrepreneurial intention (β = 0.50, *p* < 0.001).

To further test Hypotheses 4a and 4b, Model 3 removed the mediator (entrepreneurial alertness) from the initial model. The results of this comparison are shown in **Table 4**.

**Table 4:** Comparison of structural equation models (Models 2 and 3).

Variables	Model 2		Model 3
		
	Entrepreneurial alertness	Entrepreneurial intention	Entrepreneurial intention
Creativity	0.45^∗∗∗^	0.01	0.28^∗∗∗^
Proactive personality	0.51^∗∗∗^	-0.12	0.20^∗∗∗^
Entrepreneurial alertness	-	0.60^†^	-


**N* = 735.*

*Model 2: as in **Table 2**.*

*Model 3: creativity + proactive personality→ entrepreneurial intention (without mediator).*

*^†^Marginal significant.*

*^∗∗∗^*p* < 0.001.*

In **Table 4**, Model 3 shows that creativity has direct and significant effects on entrepreneurial intention (β = 0.28, *p* < 0.001), meanwhile, proactive personality has direct and significant effects on entrepreneurial intention (β = 0.20, *p* < 0.001). Interestingly, these effects become insignificant when the mediator is taken into the model. The values of β in Model 2 suggest that creativity (with β = 0.01) and proactive personality (with β = -0.12) are irrelevant with the entrepreneurial intention if entrepreneurial alertness performs as mediator.

## Discussion and Conclusion

The current research empirically supported Hypothesis 1, Hypothesis 2, and Hypothesis 3. The estimated path coefficients of our structural equation model confirm the positive effects of creativity and proactive personality on entrepreneurial alertness, and thus support the Hypotheses 1 and 2. Moreover, Hypothesis 3 is also proved by the positive and significant coefficient of entrepreneurial alertness on entrepreneurial intention (see **Figure 2**). A further comparison between models with and without mediator suggests that, the effects of the independent variables (creativity and proactive personality) on the dependent variable (entrepreneurial intention) have been mediated completely by the mediator (entrepreneurial alertness). Hence, Hypotheses 4a and 4b are further confirmed.

Although rich, the literature on entrepreneurial intentions has neglected two important items: (1) the paths from creativity and proactive personality to entrepreneurial alertness and (2) the mediation effect of entrepreneurial alertness on entrepreneurial intention. Drawing on a nationally representative survey, this study has filled this gap in the literature and provided evidence of the combined effects of creativity and proactive personality on entrepreneurial alertness and entrepreneurial intention. This study also suggests that creativity, proactive personality, and entrepreneurial alertness are three key factors in the entrepreneurship process.

Most fundamentally, we have found that the proactive personalities and creativity of students are positively related to their entrepreneurial alertness, which in turn influences entrepreneurial intention. As predicted, our study demonstrated that the effects of creativity and proactive personality on entrepreneurial intention were mediated through entrepreneurial alertness. The mediation model showed that the students who scored higher in creativity and proactive personality tended to have stronger entrepreneurial intention. This can be ascribed to the contribution made by perceived entrepreneurial alertness during the formation of entrepreneurial intention. This study not only reconfirms the common view that the alertness to opportunities is a cognitive characteristic, but also confirms that creativity and a proactive personality manifest themselves in personal entrepreneurial intention through mediating processes and mechanisms. In addition, as Zampetakis (2008) has noted, “to date, however, researchers have not examined the central theoretical role creativity might play in explaining the relationship between proactive behavior and entrepreneurial intentions.” The current study argues that important role of creativity must not be neglected when we study the effects of cognitive variables on entrepreneurial intentions. This study demonstrates the influence of creativity and proactive personality on entrepreneurial intentions. It extends the current entrepreneurial alertness literature by addressing their mediating effect on the relationship between personal traits and entrepreneurial intention.

Another important contribution of current study lies in improving the understanding of the importance of entrepreneurial alertness and its related mechanisms. Most alertness studies have focused on identifying the antecedents, rather than the outcomes, of alertness. Since little research has been carried out on the consequences of alertness, the value of present study may relate to the fact that it confirms that entrepreneurial alertness is positively and significantly related to entrepreneurial intention. In other words, it is valid to state that there is a direct path from entrepreneurial alertness to entrepreneurial intention (McMullen and Shepherd, 2006). The present findings show that entrepreneurial alertness is a vital factor to consider when exploring the way in which personal traits influence entrepreneurial intentions. It contributes to the field of entrepreneurship research by showing that cognitive variables play a crucial role in developing theories related to the entrepreneurial process (Hermann et al., 2007).

This study is particularly significant because it traces the link between personal traits and intention in the context of a non-Western culture. Given that most studies of entrepreneurial alertness and entrepreneurial intention have been carried out in Western countries, in particular, the United States, our findings are noteworthy in empirically confirming that entrepreneurial alertness is associated with intention in a non-United States culture. These results also show that the college students with higher entrepreneurial alertness tend to exhibit stronger entrepreneurial intentions in mainland China, supporting previous theoretical conclusions in a range of different contexts. In effect, it shows that McMullen and Shepherd’s (2006) results can be replicated in an international context.

The present study has some practical implications, as it can help policy makers, university administrators, and teachers to design and implement relevant interventions to enhance students’ entrepreneurial intentions. In particular, this study points to key antecedents of entrepreneurial alertness. As it has been argued that alertness can be developed and cultivated (Baron, 2004); this reinforces the idea that entrepreneurship education should focus not only on the technical aspects of entrepreneurship, such as business planning, but also on personal traits (Heinonen and Poikkijoki, 2006; Zampetakis, 2008). As creativity and proactive personality are crucial components of the entrepreneurial process, it is also essential to cultivate creativity and positive personality traits. In China, entrepreneurship education must move beyond traditional examination-oriented teaching styles to focus on promoting positive personal traits, such as a proactive personality and creativity. Given the potential value of creativity and a proactive personality, university training programs could be designed to facilitate cognitive processes involving entrepreneurial alertness (Solesvik et al., 2013), to enhance university students’ entrepreneurial orientation, particularly in relation to building their own businesses (Zampetakis, 2008). The policy interventions need thus to be targeted so as to encourage the college students to start their own business.

### Limitations

Before closing this section, some potential limitations shall be addressed of the analysis. Although a large-scale survey was carried out to guarantee the representativeness of the sample, field surveys of this type are highly dependent on self-reported questionnaires. This can lead to common method variance; as a result, the co-variance between the explanatory variables and the explained variable could not be eliminated. Although the internal consistency of our raters was high, the ratings are subjective. In future, researchers could consider longitudinal studies to further confirm which individuals become successful entrepreneurs and what kinds of cognitive feature significantly contribute to their entrepreneurial behavior. Other important variables, such as risk preference and entrepreneurial passion, as well as control variables such as prior entrepreneurial education and experience could be highlighted in studies of entrepreneurial intention.

## Author Contributions

RH designed the work. RH and WZ designed the field survey. LW assisted in the data collection. RH, WZ, and PB analyzed the data and experiment result. LW assisted in the data analysis. RH and PB wrote the manuscript.

## Conflict of Interest Statement

The authors declare that the research was conducted in the absence of any commercial or financial relationships that could be construed as a potential conflict of interest.
